# Successful Treatment of Abdominal Wall Advanced Endometriosis-Associated Clear Cell Carcinoma with AKT Pathway Inhibitor: Case Report

**DOI:** 10.3390/medicina60121946

**Published:** 2024-11-26

**Authors:** Ya-Ting Ko, Ching-Hsuan Wu, Cheng-Shyong Chang, De-Wei Lai, Ta-Chih Liu

**Affiliations:** 1 Department of Hematology-Oncology, Chang Bing Show Chwan Memorial Hospital, Changhua 505029, Taiwan; 2 Department of Molecular Biology and Cell Research, Chang Bing Show Chwan Memorial Hospital, Changhua 505029, Taiwan; 3 Department of Medical Research, Taichung Veterans General Hospital, Taichung 407219, Taiwan

**Keywords:** endometriosis-associated clear cell carcinoma, next-generation sequencing, alpelisib, everolimus

## Abstract

The emergence of endometriosis-associated clear cell carcinoma (CCC) within the abdominal wall is a notably rare phenomenon. This condition predominantly impacts females who have previously undergone surgical interventions, including hysterectomy or caesarean section (C-section), with the malignant transformation of endometriosis within the post-surgical abdominal scar posited as a likely mechanism. Herein, we delineate a distinctive case of endometriosis-associated CCC emanating from the abdominal wall. The therapeutic approach for the patient encompassed surgical resection, complemented by a regimen of adjuvant chemotherapy, radiotherapy, immunotherapy, and targeted therapy. Despite these measures, the patient experienced disease progression, manifested by bilateral inguinal lymph node involvement and metastasis to the left femoral bone. Advanced molecular diagnostics, specifically next-generation sequencing (NGS) of the resected specimen, identified a targetable *PIK3CA E726K* mutation. Subsequent treatment with alpelisib and everolimus was initiated, culminating in a sustained complete response.

## 1. Introduction

Endometriosis-associated clear cell carcinoma (CCC) arising within the abdominal wall is rare. It usually affects women who have undergone operations such as hysterectomy or caesarean section (c-section), with a probable explanation being the malignant transformation of endometriosis within the abdominal scar [1]. The incidence rate of abdominal wall endometriosis is observed in 0.03% to 1.08% of women after c-section or hysterectomy. Malignant transformation has been reported in approximately 1% of endometriosis cases [1,2,3]. The endometriosis-associated cancers include endometrioid carcinoma (70%), sarcoma (25%) and clear cell carcinoma (5%).Thirty-nine cases of CCC arising from abdominal wall endometriosis have been reported in the literature between 1986 and 2023; the average age at diagnosis was 45.7 years (ranges from 37 years to 60 years), and most cases had a history of previous c-section. The literature review showed that the average follow-up time was around 15 months, and about 25% of women died within 15 months of diagnosis. Here, we report a case of endometriosis-associated CCC arising from the abdominal wall. 

## 2. Case Presentation

**Figure 1:**
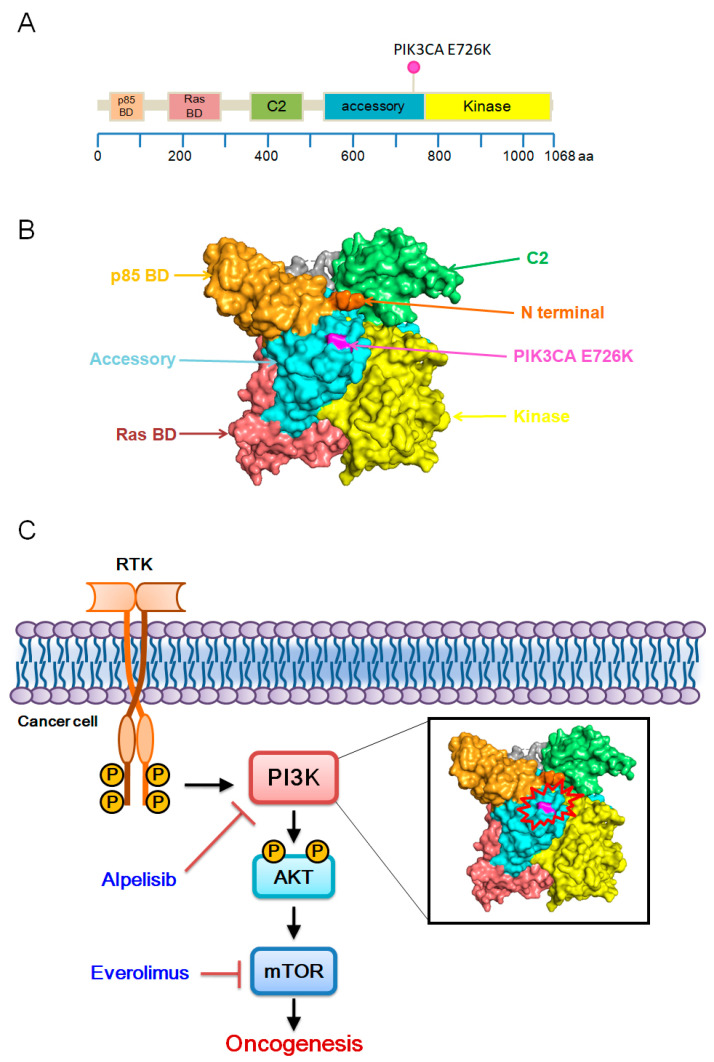
Overall structure of PI3K class 1. (**A**) Domain organization of PI3Ks. p85 BD, p85 alpha binding domain (amino acids 32–108); Ras BD, Ras binding domain (amino acids 174–291); C2, C2 domain (amino acids 351–483); accessory, accessory domain (amino acids 520–703); kinase, kinase core domain (amino acids 798–1014). The color pink indicates *PIK3CA E726K* mutation site. Amino acids 1–1068 of PIK3CA E726K are described with annotated mutations from the following website: https://www.oncokb.org/ accessed on 22 September 2024. (**B**) Surface representations of PI3Kα architectures (PDB 7PG5) (**C**) PI3K/AKT/mTOR pathway.

The patient is a 50-year-old woman with no previous history of cancer who presented with a palpable mass in her lower abdomen for 3 months. Her medical history included three C-sections (29, 27, and 24 years ago, respectively). There was no reported family history of cancer. She had initial presentation of a palpable lower abdominal wall mass that progressively increased in size with periodic pain for 6 months. She visited a regional teaching hospital where abdominal CT was performed and revealed an abdominal wall cystic and solid lesion (5 cm × 4 cm × 3 cm). On 4 June 2019, the patient underwent a laparoscopic biopsy of the abdominal wall tumor, and pathology revealed endometriosis-associated clear cell carcinoma of the abdominal wall. Then, she came to a medical center for second opinion on 17 June 2019. Gynecologic ultrasonography was carried out and showed no obvious lesion over the endometrium or uterus. The following PET-CT scan showed an FDG avid mass around the left rectus abdominis muscle, uterine cavity, and high rectal region. She was scheduled for three courses of neo-adjuvant chemotherapy with paclitaxel, cisplatin, and doxorubicin, experiencing two episodes of neutropenic fever. However, follow-up abdominal CT revealed stable disease. Subsequently, she underwent open surgery with wide resection of the abdominal wall tumor on 6 September 2019. No other tissues were taken. Staging: Stage IVB, TxN0M1; FIGO stage IVB.The tumor mass measured 6.2 × 4.5 × 3 cm with margins as close as 0.1 cm in some foci. However, follow-up CT imaging on 21 February 2020, showed progressive enlargement of bilateral inguinal lymph nodes. The initial CT scan was performed on 30 May 2019. During the treatment course, CT scans are repeated every 3 months. After achieving complete remission (CR), scans are conducted every 6 months for 3 years and then annually until the 5-year mark. On 26 February 2020, the patient underwent bilateral inguinal lymph node excisional biopsy, revealing metastatic adenocarcinoma in one of four lymph nodes from the right inguinal area and one of three lymph nodes from the left inguinal area. Pembrolizumab immunotherapy (200 mg) was administrated every 3 weeks for 15 cycles until 3 June 2022. During immunotherapy, abdominal CT showed left inguinal lymph node enlargement, prompting 33 fractions of external beam radiation therapy (6600 rads) from 9 August to 29 September 2021. Due to disease progression and further left inguinal lymph node enlargement, the patient was transferred to our hospital for additional treatment in June 2022. A PET scan was performed on 22 June 2022, revealing multifocal hypermetabolic foci, with the most intense located in the left inguinal lymph node and left upper femoral bone. Targeted amplicon-based NGS conducted in a Clinical Laboratory Improvement Amendments–certified laboratory identified *PIK3CA* E726K, *SMARCB1* R377C, and *TERT* promotor −124 C>T mutations. Nucleic acid extracted from samples were libraried with commercial library kit (Thermo Fisher Scientific, Waltham, WA, USA). Prepared libraries were loaded onto an ion chip automatically using the ion chef (Thermo Fisher Scientific). Sequencing was performed with the Ion S5™ XL Sequencer. The sequencing data were automatic mapped through Torrent Suite™ (Thermo Fisher Scientific) and automated analysis was conducted with Ion Reporter™ Software (v. 5.16). Mutations were further analyzed according to OncoKB™ reports. According to OncoKB™ reports [4], *PIK3CA* mutations are most prevalent in endometrial cancer, with an altered frequency close to 40%, including 103 out of 1386 mutation sites, such as E726K (Figure 1A,B). Nevertheless, the oncogenic and mutation effect of E726K remain inconclusive. A multidisciplinary oncology team decided on treatment with alpelisib (a PI3K inhibitor) and everolimus (an mTOR inhibitor), targeting the PI3K/AKT/mTOR pathway (Figure 1C). Taken together, the patient underwent surgical excision followed by adjuvant chemotherapy, radiotherapy, immunotherapy, and target therapy. However, disease progression occurred with bilateral inguinal lymph nodes and left femoral bone metastases. Next-generation sequencing (NGS) of the excisional sample revealed a druggable PIK3CA E726K mutation. The patient was treated with alpelisib and everolimus, achieving a durable complete response.

**Figure 2:**
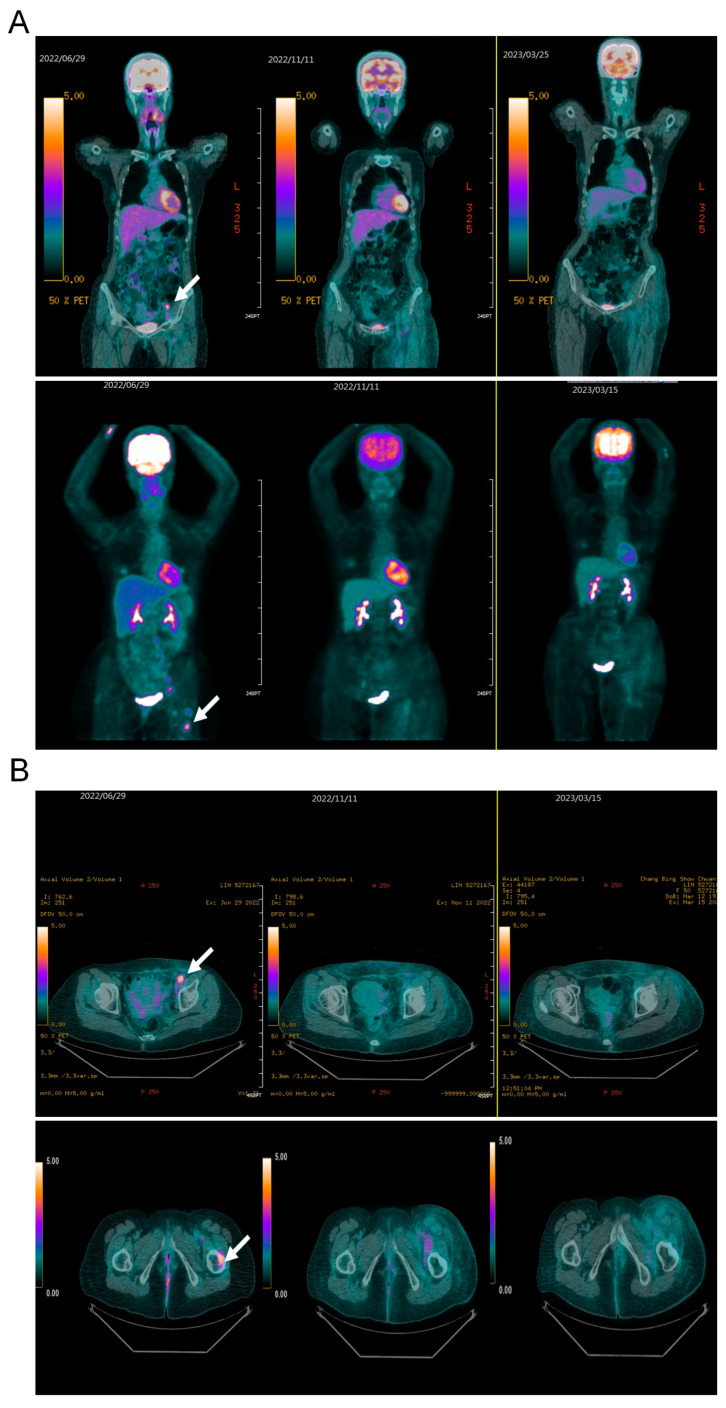
Response to treatment in a patient with endometriosis-associated clear cell carcinoma, presenting with left inguinal lymph node and left femoral bone metastases. (**A**) 3D maximum intensity projection of 18F-FDG-PET scans (anterior views) conducted before treatment (June 2022), 5 months after treatment initiation (November 2022), and 9 months into treatment (March 2023). The pretreatment images show multifocal hypermetabolic foci (indicated by arrows), with the most intense and notable activity in the left inguinal lymph node and left upper femoral bone. (**B**) A selected transaxial slice showing increased FDG uptake in the left inguinal lymph node and left upper femoral bone.

After 3 months of treatment, the patient experienced manageable side effects including watery diarrhea, hyperglycemia, and hyperlipidemia. PET-CT revealed a complete response which persisted for 24 months (Figure 2A,B).

## 3. Discussion

To our knowledge, this is the first case showing a sustained complete response in endometriosis-associated CCC treated with alpelisib and everolimus. In this case, the combination therapy led to durable disease control for over 2 years. The Akt signaling pathway is activated by receptor tyrosine kinases, which induce the production of phosphatidylinositol (3,4,5)-trisphosphates (PIP3) by phosphoinositide 3-kinase (PI3K). These PIP3 phosphorylate Akt at Thr308, leading to partial activation, while phosphorylation at Ser473 by mTORC2 completes full Akt activation. The activation of the PI3K/Akt pathway is implicated in various cancers [5,6]. 

In fact, numerous whole-exome sequencing studies have shown that many *PIK3CA* mutations found in normal uterine and endometriotic epithelium are non-silent and are typically consistent with oncogenic mutations [7]. At the same time, the most common genetic alterations in endometriosis-associated CCC affect the KRAS/PI3K pathway. Therefore, these *PIK3CA* mutations may have significant functional relevance and could represent the first step in the progression of endometriosis-associated cancer [8]. The theory proposed by Anglesio is consistent with this, demonstrating that in cases of ovarian cancer associated with endometriosis carrying activating *PIK3CA* mutations, the same mutations repeatedly appear in both cancer and endometriotic specimens [9]. This may pave the way for subsequent genetic events leading to carcinogenesis. The *PIK3CA* encodes p110α, the catalytic subunit of class 1A PI3K, and its aberrant activation stimulates cellular proliferation, survival, and migration, thereby driving cancer formation and progression [5]. Alpelisib selectively inhibits the p110α subunit of PI3Kα and is approved for treating breast cancers with *PIK3CA* mutations [10]. Alpelisib is a tier 2 drug for tumors other than breast cancer. Everolimus (C53H83NO14) is an effective anti-cancer drug for human breast cancer [11], inhibiting the growth and aggressiveness of breast cancer cells through the PI3K/AKT/mTOR pathways [12]. Based on this rationale, we treated the patient with a combination of alpelisib and everolimus, achieving long-term disease control. Immunotherapy may benefit patients with MMR-deficient clear cell endometrial cancer [13], but in this case, the absence of MMR deficiency likely contributed to the lack of response to immunotherapy. 

However, the patient experienced adverse effects from alpelisib, including grade 3 hyperglycemia, grade 3 watery diarrhea, and grade 2 hypercholesterolemia, during the course of treatment [10]. Due to these side effects, the alpelisib dosage was reduced to 50% of the original dose, and the patient was treated with oral hypoglycemic agents and antidiarrheal medications. After recovery from the side effects, the alpelisib was escalated back to the original dose, and the patient achieved a sustained complete response. 

## 4. Conclusions

Our successful treatment with a combination of AKT pathway inhibitors in endometriosis-associated CCC harboring a PIKCA mutation may provide an effective treatment option for other types of cancer.

## Data Availability

Data are available upon request.
